# Between “Medical” and “Social” Egg Freezing: A Comparative Analysis of Regulatory Frameworks in Austria, Germany, Israel, and the Netherlands

**DOI:** 10.1007/s11673-021-10133-z

**Published:** 2021-11-16

**Authors:** Nitzan Rimon-Zarfaty, Johanna Kostenzer, Lisa-Katharina Sismuth, Antoinette de Bont

**Affiliations:** 1 Department of Medical Ethics and History of Medicine, University Medical Centre Göttingen, Humboldtallee 36, 37073 Göttingen, Lower Saxony Germany; 2 Department of Human Resource Management Studies, Sapir Academic College, D.N. Hof Ashkelon, 7915600 Hof Ashkelon, Israel; 3 Erasmus School of Health Policy & Management, Erasmus University Rotterdam, Rotterdam, P.O. Box 1738, 3000 DR The Netherlands

**Keywords:** Social egg freezing, Medical egg freezing, Regulatory boundary–work, Regulation analysis, Cross-national comparison, Austria, Germany, Israel, The Netherlands

## Abstract

Egg freezing has led to heated debates in healthcare policy and bioethics. A crucial issue in this context concerns the distinction between “medical” and “social” egg freezing (MEF and SEF)—contrasting objections to bio-medicalization with claims for oversimplification. Yet such categorization remains a criterion for regulation. This paper aims to explore the “regulatory boundary-work” around the “medical”–”social” distinction in different egg freezing regulations. Based on systematic documents’ analysis we present a cross-national comparison of the way the “medical”–”social” differentiation finds expression in regulatory frameworks in Austria, Germany, Israel, and the Netherlands. Findings are organized along two emerging themes: (1) the definition of MEF and its distinctiveness—highlighting regulatory differences in the clarity of the definition and in the medical indications used for creating it (less clear in Austria and Germany, detailed in Israel and the Netherlands); and (2) hierarchy of medical over social motivations reflected in usage and funding regulations. Blurred demarcation lines between “medical” and “social” are further discussed as representing a paradoxical inclusion of SEF while offering new insights into the complexity and normativity of this distinction. Finally, we draw conclusions for policymaking and the bioethical debate, also concerning the related cryopolitical aspects.

## Introduction

Technological innovation makes it possible to harvest and cryopreserve unfertilized eggs (oocytes), store them, and use them later in life through assisted reproductive technologies (ART). The procedure of egg freezing generally includes two main stages: In the first stage, oocytes are retrieved and cryopreserved. In the second stage, when wishing to use the eggs for conception, the frozen eggs are thawed, fertilized (using IVF techniques), and finally transferred back into the uterus.

When first developed as an experimental procedure in the late 1980s (Waldby 2015), egg freezing was mainly used for “medical” reasons—what has been referred to as medical egg freezing (i.e., as a protective measure against adverse medical treatment outcomes, e.g., diminished fertility caused by oncology treatment). However, the development of vitrification (“fast freezing”) and intracytoplasmic sperm injection (ICSI) techniques led to a renewal of interest in egg freezing in the early to mid-2000s (Shkedi-Rafid and Hashiloni-Dolev 2011). In 2012, both the American Society of Reproductive Medicine (ASRM & SART 2013; ASRM 2018) and the European Society of Human Reproduction and Embryology (ESHRE 2012) lifted the experimental label of the procedure. Therefore, egg freezing has been endorsed as a mean for preserving fertility due to so called “social” reasons—what has been recognized as non-medical or “social” egg freezing.

Empirical research from Israel, Turkey, the United Kingdom, and the United States shows that “social” reasons include primarily relationship factors (e.g., singlehood), economic factors (e.g., lacking financial resources), career plans, and other reasons (Kılıç and Göçmen 2018; Inhorn et al. 2018; Baldwin et al. 2018).

Egg freezing in general and for “social” reasons in particular has led to heated debates in healthcare policy and practice and related social and bioethical debates. The discussion is shaped by arguments related to genetic linkage, motherhood and family, reproductive control and autonomy, (bio) medicalization, success rates limitations, the health risks involved, empowerment, alienation, and the biological boundaries which may (or may not) be crossed (ESHRE 2012; Pennings 2013; Bernstein and Wiesemann 2014; Robertson 2014; Dondorp and De Wert 2009; Kostenzer et al. 2021a; Kostenzer et al. 2021b).

As much as the technology is controversial and the discussion is diverse, so is its terminology. It ranges from non-medical or social egg freezing (Baldwin et al. 2015), freezing oocytes by healthy women (Pennings 2013), elective egg freezing (Inhorn et al. 2018), to oocyte cryopreservation for age-related fertility loss (ESHRE 2012), or planned oocyte cryopreservation (ASRM 2018). The different terminologies also reflect the controversy around whether there is a type of medical need even when it comes to non-medical egg freezing. In this paper we use the terms “medical egg freezing” (MEF) and “social egg freezing” (SEF) as those are the most commonly used terms.

Aside from issues related to SEF as such, another crucial issue to be considered concerns the distinction between MEF and SEF. Do reasons behind egg freezing qualify for a categorization into “medical” and “social”? If so, how can a distinction between the two be made in practice?

What can be considered a medical reason or need is difficult to determine and is differently negotiated among healthcare professionals, ethicists, policymakers, and patients. The debate revolves around whether or not age-related fertility decline should be regarded as a medical condition justifying the use of medical means—as a form of preventive medicine (Borovecki et al. 2018). Critics oppose this justification by considering SEF as (bio)medicalization of social problems (Dondorp and De Wert 2009), claiming SEF in fact uses medical technology for “solving” a nonmedical “problem” (Petropanagos et al. 2015, 668). Faced with socio-economic pressures and gendered labour-market barriers, women nowadays tend to postpone childbearing and are thus faced with loss of fecundity. Under such circumstances, SEF can be perceived as an individual medical solution to a wider social problem, which should thus be dealt with through social solutions; i.e., cultural accommodations, policy measures, and public health approaches (Lemoine and Ravitsky 2015).

Van de Wiel (2015) notes in this context that the differentiation into medical and non-medical oversimplifies the complexity of the issue. She challenges this categorization and its usage for regulatory decisions. Pennings (2013) argues that the term “social” creates a notion of desire rather than need. He further claims this differentiation is blurred and should hence not be made.

Yet in practice, the categorization into “medical” and “social” is still being made and remains a main criterion for regulating access to and cost coverage of egg freezing.

The European IVF-monitoring Consortium (EIM) for the ESHRE, as well as its Working Group on Oocyte Cryopreservation, investigated the regulation of oocyte cryopreservation in forty-three countries, showing a differentiation between the indications for egg freezing (Calhaz-Jorge et al. 2020; Shenfield et al. 2017). It was found that legislation is diverse and funding systems are highly variable. While such studies provide an important overview regarding the regulatory conditions for egg freezing, we aim at taking a step further by providing an in-depth critical analysis of regulatory frameworks of selected countries.

The aim of this paper is hence to explore how the differentiation between what is considered “medical” and “social” finds expression in regulatory frameworks and the way those may vary across countries. Based on empirical study, we illustrate the normative and constructed nature of the categorization into “medical” and “social” (see, e.g., Kingma 2013). Relying on the assumption that rules and regulations are being set as mechanisms of boundaries demarcation in actual practice (Zarhin et al. 2018), we therefore draw in this context on what was termed by Zarhin et al. (2018) as “regulatory boundary-work”; in our case: if and how the regulatory frameworks define and differentiate between MEF and SEF. Within this context, potential practical boundaries may be set in relation to age limitation, familial/marital status, health insurance type, and medical indications. Such regulatory boundaries defining legal limitations and access can in practice play an important role in people’s reproductive and life choices. Indeed, ART and egg freezing often raise equity concerns, as they may not be equally accessible to all women (Lemoine and Ravitsky 2015). While within the scope of this study we cannot focus on the boundary-work itself (i.e., the actual considerations and processes involved in setting the regulation), we aim at analysing the work these boundaries create (i.e., in the form of meanings and possible implications).

In order to better examine this boundary-work we chose to focus on four countries: Austria, Germany, Israel, and the Netherlands. While all four are at the cutting edge of medical technologies and care standards, they represent a diverse spectrum of regulatory frameworks in the context of ART as well as generally differentiated professional cultures in biomedicine. Therefore, our choice of countries resulted in a diverse, yet balanced, comparison highlighting a wide spectrum of regulatory frameworks that is of value for understanding the boundary-work in other countries as well. By conducting a cross-national comparison of regulatory frameworks, we explore the diverse regulatory and funding schemes reflecting different negotiation and interpretations of egg freezing as medically necessary or elective treatment.

## Methodology

Our research draws inspiration from the “decentred comparative research approach” (Wrede et al. 2006), which acknowledges the need for a context sensitive analysis, while emphasizing the importance of cross-national comparison in healthcare research. Drawing on collaboration across research nationals and cultures, the decentred comparative approach facilitates the uncovering of the social situatedness of healthcare as a study object—allowing researchers to understand the ways healthcare systems and practices are situated in time and place. It is further a highly (self) reflexive approach taking into account the hazard of ethnocentrism and the ways our own knowledge and perceptions as researchers are socially situated (ibid).

Our research team was formed during 2018-2019 and consists of four members—internationally dispersed with in-depth familiarity of the countries concerned: Austria, Germany, Israel, and the Netherlands. Our heterogeneous team employed both socially situated and socially distributed inter-disciplinary expertise, including diversity in nationality, ethnicity, religious background and scientific background, enabling a decentred analysis. The team’s (inter)disciplinary background includes expertise in sociology, bioethics, health policy, and medicine. The cooperation allowed for both a close reading and informed analysis of the regulation, as well as normative insights from a sociocultural and moral perspective. While collaborating towards the common aim, we constantly challenged each other’s understandings and interpretations by discussing different perspectives. This process aimed at understanding the ways both MEF and SEF are regulated and therefore understood and practiced in the different sociocultural contexts. For this purpose, we defined regulations as official documents including legislation, ministerial decisions and memorandums, medical guidelines, medical and (bio)ethical recommendations, relevant position papers, and related professional committees’ reports. Documents concerning funding regulations vary between countries and include ministerial memorandums (in the form of secondary legislations), coverage policy documents, and insurance regulations.

We used broad inclusion criteria, collecting not only documents which specifically deal with egg freezing but also documents dealing with related procedures (e.g., IVF) which are of relevance for the specific regulatory framework. In addition, we collected and analysed not only legally binding regulatory documents but also recommendations, position papers, and guidelines published by professional (medical or (bio)ethical) associations. In countries in which the official legal framework is missing or fragmented, these documents hold a significant role in the regulatory boundary work. Their role, however, should not be neglected also in countries with clear legislation (i.e., in inducing and shaping formal legislation). While we are aware of the distinct differences in nature and implications of these documents, they together form the regulatory framework and shape the handling of egg freezing.

Our empirical work followed a systematic approach, divided into three phases. (1) We collected the relevant documents and conducted a thorough analysis. Documents were collected following an extensive archive (including online) research and literature analysis. When needed, translations into English were made to allow for better understanding and comparison. All translations (from German, Dutch, and Hebrew into English) were conducted by the research team. (2) We prepared summary reports for each country, containing a detailed overview of the up-to-date regulations and its preliminary analysis. These reports also contained the relevant academic and public debates as presented in scientific literature and media accounts. The reports were circulated among the research team and extensively discussed in several digital and in-person meetings for critical feedback and preliminary comparison. The first two phases were accompanied by consultations with relevant experts in the fields of law, social sciences, ethics, and medicine in all four countries to ensure full coverage of the relevant regulatory framework. (3) In the final phase, we conducted our comparative analysis in a systematic manner, which generally corresponds with the qualitative content analysis method (Weber 1990). Summary reports were analysed thematically and compared cross-nationally to uncover discursive themes and emerging codes (Denzin and Lincoln 1994). This enabled us to detect different constructions of MEF and SEF which could be further interpreted as reflecting different forms of boundary-work.

## Background: Relevant Legislation and Official Regulation

### Austria

In Austria, assisted reproductive medicine is primarily regulated under the Law on Reproductive Medicine (FmedG “Fortpflanzungsmedizingesetz” 1992) and the IVF Fonds Act (1999). The FMedG was introduced in 1992 and amended in 2015 (FMedRÄG “Fortpflanzungsmedizinrechts-Änderungsgesetz” 2015). The amendment represented a shift from a rather restrictive to a more permissive regulatory approach (Flatscher-Thöni and Voithofer 2017, 47), for example, in allowing altruistic egg donation, making ART accessible to female same-sex couples, and legalizing prenatal genetic diagnosis. Singles are, however, excluded from accessing ART. The IVF Fonds Act regulates related processes and coverage of the use of ART, that is, IVF and ICSI, both of relevance when it comes to the usage of previously frozen eggs.

In line with the FMedRÄG, egg freezing is only allowed on medical grounds. The quality assurance of the cryopreservation of gametes, ovarian tissue, and testicle tissue is regulated under the Tissue Safety Act (2018).

### Germany

In Germany, the regulatory framework of reproductive technologies is shaped by the German Embryo Protection Act (EPA) (1990), which has been identified as rather restrictive as it prohibits the usage of several reproductive technologies (e.g., egg donation). The law also sets a unique limitation by only allowing the freezing of fertilized eggs at the pronuclear state (Robertson 2014), while limiting the creation and the cryopreservation of surplus embryos (Möller and Makoski 2020). The freezing of unfertilized eggs is, however, not legally restricted and thus takes place for both medical and non-medical reasons.

### Israel

In Israel, the usage and funding of ART are regulated under the Israeli National Health Insurance Law (1994). The legislation enables the accessibility and funding of many of the technologies (including IVF). While some ARTs are regulated via directives issued by the Israeli Ministry of Health, others (e.g., egg donation) are regulated by specific laws. The Israeli Ministry of Health issued two directives—devoted specifically to the regulation of egg vitrification. The first directive (Israeli Ministry of Health 2010) declared that vitrification should no longer be considered an experimental technology. The following directive (Israeli Ministry of Health 2011a) added an elaboration of the indications and conditions justifying the use of egg freezing. Following these directives, both MEF and SEF are allowed and take place.

### The Netherlands

The use of ART is closely regulated by both law and code of practice in the Netherlands (Shenfield et al. 2017). Of specific relevance to egg freezing are the Artificial Fertilization Donor Data Act, containing rules for storage, management, and provision of data from donors in the event of artificial donor fertilization (Wet donorgegevens kunstmatige Bevruchting 2002), the Embryo Act, regulating the handling of germ cells and embryos (e.g., prohibiting cloning) (Embryowet 2002), and the IVF decree covering all aspects of the IVF trajectory (Planningsbesluit in-vitrofertilisatie 2016). In April 2011, a majority in the Second Chamber supported the legalization of egg freezing for medical as well as non-medical reasons (together with the possibility for egg donation). However, the number of ART centres is limited by law and only licensed healthcare organizations may provide egg freezing (Calhaz-Jorge et al. 2020).

## Findings: A Comparative Analysis of Regulatory Frameworks of Egg Freezing

Our analysis revealed two main themes stemming from the regulatory frameworks in the four countries: (1) a regulatory definition of MEF and its distinctiveness in relation to social motivations for using egg freezing; and (2) a hierarchy of these motivations. Yet, within each of these shared themes we detect cross-national differences.

For a comparative overview of the regulatory framework in the four counties see table 1.

**Table 1:** Comparative overview of egg freezing regulations

Country	Regulation	Freezing	Coverage	Definition	Age limits	Usage for Reproduction
Austria	FMedG, FMedRÄG, IVF Fonds-Act, TSA, medical guidelines	MEF only	None (MEF: medication may be covered by the insurer)	Diagnosis-based (not elaborated) MEF includes oncological diagnosis	Only regarding IVF *funding:* maximal age of 40 (women) and 50 (men)	Partial coverage of IVF for medically diagnosed couples (70% of up to 4 cycles of IVF)
Germany	EP Act, SGB V §27a, G-BA guideline, medical guidelines	MEF and SEF	MEF yes (including medication and preservation) SEF no	MEF: medical indication (limited elaboration) MEF clearly includes oncological diagnosis SEF: others	For MEF *funding* limitations: Retrieval until the age of 40 For IVF *funding:* 25–40 (women); 25–50 (men)	Partial coverage of IVF for medically diagnosed married couples (50% of up to 3 cycles of IVF)
Israel	National Health Insurance Law Ministry directives- Secondary legislation	MEF and SEF SEF: *usage* limitations: Up to 4 cycles or 20 retrieved eggs	MEF yes (including medication and preservation until the birth of two children) SEF no	MEF: medical indications (very elaborated) MEF clearly includes oncological diagnosis as well as medically diagnosed fertility decline SEF: others	For MEF *funding* limitations: Retrieval until the age of 39 Duration of the preservation- until the age of 42 For SEF *usage* limitations: Retrieval 30–41. Eggs usage up to the age of 54 For IVF *funding:* 18– 45 (women)	Full coverage of IVF for medically diagnosed couples and single women (until the birth of two children; all marital statuses)
The Netherlands	Artificial Fertilization Donor Data Act, Embryo Act, IVF decree, Ministry decisions, medical guidelines and code of practice	MEF and SEF	MEF yes (including medication and preservation) SEF no	MEF: medical indications (elaborated) MEF clearly includes oncological diagnosis as well as medically diagnosed fertility decline SEF: others	For SEF *usage* limitations: Retrieval until 40, eggs usage up to the age of 50 For IVF *funding:* maximal age of 43 (women)	Partial coverage of medically diagnosed women (Full coverage of 3 IVF cycles; all marital statuses)

### Defining Medical Egg Freezing and its Distinctiveness

One main common theme emerging from our analysis is that all four regulatory frameworks attempt at defining MEF and distinguishing between MEF and SEF. However, they differ in the extent to which they present such a distinction, as well as in the definitions and indications they use for creating it.

#### Austria

In the Austrian case, where SEF is not allowed, the distinction between MEF and SEF is crucial. Following the Amendment of the Austrian Law on Reproductive Medicine (FMedRÄG) in 2015, the harvesting and storage of reproductive cells is only allowed on medical grounds. Furthermore, the current regulation excludes singles from accessing ART in general, which therefore also explains the restrictive approach towards SEF in the country (Kostenzer 2020).

Medical ground is defined in the legislation as serious risk of not achieving pregnancy through sexual intercourse in case of “physical suffering” or its treatment. Specific medical indications, however, are not listed in the FMedG. Therefore, while in principle an important distinction is made between the “medical” and the “social,” the legislation does not provide any clear or practical demarcation lines, leaving room for the negotiation and the decision of medical professionals.

Clearer indication of what may count as “medical” can be found in the IVF Fonds Act, regulating the funding of IVF treatments (which also qualify for a partial coverage of the egg freezing procedure):

1. sterility of the woman originating from issues related to fallopian tube, endometriosis or polycystic ova syndrome;
2. and/or infertility of the man;

Another source for understanding the criteria for medical indications are relevant medical treatment guidelines. The Austrian Association for Gynecology and Obstetrics jointly published guidelines on fertility preservation during oncological treatment with their German and Swiss counterparts (OEGGG, DGGG, and SGGG 2017). Within these guidelines, oncological and rheumatic conditions are acknowledged as medical indications justifying egg freezing. Another form of medical indication, outlined by the Austrian Bioethics Commission (2015) is medically diagnosed early menopause.

#### Germany

The German legislation does not restrict the performance of both MEF and SEF. However, and similar to other practices in reproductive medicine, the legislator did not systematically regulate fertility preservation, creating a grey zone and legal uncertainty (see e.g., Leopoldina 2019; Broesicke et al. 2017). Due to this lack of regulatory clarity, the German medical association published guidelines for the retrieving and transfer of human gametes, yet focusing on technical instructions (regarding freezing methods, temperatures etc.) (Bundesärztekammer 2018).

Very recently, Germany initiated new guidelines regulating the funding of MEF. In August 2021, the German Social Code was amended (SGB V §27a 2021). With the TSVG (Appointment Service and Supply Act), the entitlement of public health insurance funding in case of artificial insemination was expanded to include the cryopreservation of gamete cells and tissue in cases of threatened fertility due to disease or germ cell damaging treatments. Following an extensive evaluation process, in December 2020, the Federal Joint Committee published and adopted guidelines, which came into force in February 2021 (Gemeinsamer Bundesausschuss (G-BA) 2021a) and finally implemented in July 2021 (Gemeinsamer Bundesausschuss (G-BA) 2021b). The guidelines determine that performance and funding of MEF will be given under the following medical indications:

1. surgical removal of the gonads,
2. radiotherapy with expected damage to the gonads, or
3. potentially fertility-damaging medication

Exploring the definition of MEF and its distinction from SEF further, insurance coverage regulations initiated by the German organization of private health insurances—the PKV (Private Krankenversicherung)—prior to the new regulations, refer to oncological conditions (DGHO 2017). This reference, which is also prevalent in the new G-BA guidelines, corresponds with the above-mentioned joint guidelines published by the Austrian, German, and Swiss associations for gynaecology and obstetrics (OEGGG, DGGG, and SGGG 2017).

#### Israel

Unlike the Austrian and German frameworks, in Israel there is a detailed regulatory framework of fertility preservation. The Israeli regulation clearly differentiates between MEF and SEF. The Israeli National Health Insurance Law (1994, section 6 amended in 2011) mentions chemotherapy and radiation therapy as indications justifying fertility preservation and its funding (in one of the following methods: egg-freezing, embryo freezing, and ovarian tissue freezing) for female patients (children, adolescents, and women). Similar indications are also published as directives by the Israeli Ministry of Health (2011b). Based on the recommendations by the Israeli National Council for Gynecology, Neonatology and Genetics. In 2011, the ministry also published a directive which defines additional medical conditions under which egg freezing will be performed (Israeli Ministry of Health 2011a, 1-2):

1. When the woman/couple is undergoing fertility treatments: in case the male partner suffers from severely low sperm quality or inability to give sperm; in case the woman suffers from low reserve of eggs; severe endometriosis or abscess in the inner genitals;
2. Increased risk for early amenorrhea due to one of the following reasons: carrying of fragile X pre-mutation; existence of other indicators for early amenorrhea; women who suffer from autoimmune diseases; and women who suffer from chromosomal or other syndromes increasing the risk for amenorrhea;
3. Women about to face surgery: preventive resection of the ovary (carrier of BRCA); any other operation which may involve resection of the ovaries;

These medical identifications extend therefore the indications for medical fertility preservation beyond cancer patients, by including other conditions and procedures that pose a risk for future fertility as well as indications for infertility.

Another important source demonstrating the relatively wide spectrum of fertility related conditions included in the Israeli regulation of MEF are funding regulations (Israeli Ministry of Health, 2018, 16). These further expand the indications for medical fertility preservation publicly covered to include women with increased risk for early amenorrhea—in one or more of the following situations: (1) women with low ovarian reserve; (2) women before gonadotoxic therapy not due to malignancies; (3) candidates for surgery to remove more than one ovary. All indications that do not fall under the definition of MEF are treated as SEF.

#### The Netherlands

The Dutch ART landscape is characterized by rather specific regulations resulting from law and code of practice. Guidelines are issued by the two professional bodies: the Dutch Society of Obstetrics and Gynecology (NVOG, “Nederlandse Vereniging voor Obstetrie & Gynaecologie”) and the Association for Clinical Embryology (KLEM, “Vereniging voor Klinische Embryologie”), including, for example, the report *Vitrification of Human Oocytes and Embryos*, which recommends allowing the use of egg freezing technology for non-medical reasons (NVOG and KLEM 2010). Following the report in April 2011, a majority in the Second Chamber supported the legalization of egg freezing for medical as well as non-medical reasons (Bos, Klapwijk, and Fauser 2012, 1; Eleveld and Van Vliet 2013).

The regulatory framework also defines MEF and its distinction from SEF. In 2012, the Dutch National Health Care Institute (“Zorginstituut”) published a report devoted to the vitrification of own oocytes and its regulation. It defines the three following indications as qualifying for MEF (van der Meer and Derksen 2012):

1. Treatments with chemotherapeutic agents that pose a risk of permanent fertility impairment;
2. Radiotherapeutic treatments where the ovaries are in the radiation field and can be permanently damaged;
3. Operative treatments where both ovaries or large parts of them must be removed (on medical grounds);

The Health Care Institute further listed the following medically related additional indications as justifying egg freezing (van der Meer and Derksen 2012):

1. Carriers of fragile X syndrome, Turner syndrome or galactosemia leading to increased risk of premature ovarian insufficiency (before the 40th birthday);
2. IVF-bound fertility related indications revealed and diagnosed during an IVF attempt (if this attempt falls under the basic insurance);

The Dutch regulatory framework therefore also includes fertility problems as medical indications accounting for MEF. Here too, all motivations and applications that do not belong to the defined medical criterion are therefore considered to be SEF.

### A Hierarchy of Medical Over Social Motivations for Egg Freezing

As demonstrated so far, the different regulatory frameworks present different scopes of the concept of “medical” and the conditions regulated under it. They therefore also represent various forms of distinctions between MEF and SEF reflected in access and coverage decisions. Moving to our next common theme, we claim that such categorizations also represent a hierarchy of certain motivations over others (Martin 2010). In what follows we will demonstrate how the regulatory frameworks represent a prioritization of MEF over SEF, inter alia via regulative restrictions and related funding policies reflecting their perceived value.

#### Austria

By outlawing SEF, the Austrian legislator not only separates SEF from MEF but also clearly prioritizes MEF as the only legitimate form of egg freezing. In the process of amending the Austrian Law on Reproductive Medicine (FMedRÄG 2015), a variety of expert bodies (including the Austrian Association for Gynaecology and Obstetrics) suggested to allow also SEF (Österreichische Gesellschaft für Gynäkologie und Geburtshilfe 2014). These efforts, however, did not receive much attention by the regulators at the time.

The prioritization of MEF is also visible in the Austrian funding policy. MEF is not covered by the Austrian social health insurance as such. However, part of the costs (e.g., medication) may be covered on a case-by-case basis subjected to the decision of the insurer. According to the FmedG, where the patient has a partner (i.e., there is a couple at stake), and under the condition that official diagnosis of a medical indication as outlined above is provided, the usage of eggs previously frozen may qualify for IVF partial coverage. According to the Austrian IVF Fonds Act (1999), if a couple qualifies, 70 per cent of the IVF costs of up to four cycles of IVF treatment are covered. Treatments are covered up to the maximum age of forty (women) and fifty (men). Storage costs of frozen oocytes is covered by the patients. SEF is not allowed and therefore not funded.

#### Germany

The German regulatory framework sets no official legal restrictions on the usage of either MEF or SEF. In terms of funding, however, the new GB-A guidelines create a clear differentiation between MEF and SEF by determining medically indicated conditions under which MEF will be publicly funded. The new regulatory framework further determines that MEF funding is limited to women under the age of 40 and covers costs related to the preparation, removal, processing, transport, freezing, storage, and subsequent thawing of the frozen egg cells (Gemeinsamer Bundesausschuss (G-BA) 2021a, 2021b).

Several attempts have been made to promote reimbursement of MEF. For example, the German National Academy of Sciences Leopoldina (2019) published detailed recommendations for new legislation of reproductive medicine, suggesting that fertility preservation should be publicly funded in case of serious illness or treatment threatening fertility. It also emphasized the differences between medical and social indications. Following these attempts, and as mentioned before, the German SGB V has been amended to include funding of MEF by the public health insurance. These ongoing discussions also reflect the hierarchy between medical and social motivations.

Similarly to the Austrian case, some of the potential costs of the usage of previously frozen oocytes may be covered under the IVF funding regulation. The German regulatory framework of IVF grants financial support to diagnosed fertility patients. Funding is given to married couples between the ages of twenty-five and forty (women), and twenty-five and fifty (men). It is limited to 50 per cent of the costs, in cases of reasonable prospect of success; i.e., if a certain number of unsuccessful attempts is not exceeded—that is of three treatment cycles. Preservation costs are not covered. In a few federal states, further financial support programs are offered (Möller and Makoski 2020; SGB V §27a 2021; Leopoldina 2019).

SEF is not funded. Furthermore, the lack of any relevant regulatory framework regarding the application of the procedure led the German speaking countries FertiPROTEKT (professional-advisory) network to establish voluntary self-guidelines for SEF. According to these guidelines, fertilized eggs following cryopreservation should be implanted before the woman’s fiftieth birthday (von Wolf, Germeyer, and Nawroth 2015).

#### Israel

While both MEF and SEF are regulated and performed in Israel, funding regulations differ between the two.

Fertility preservation is fully covered (including medication costs) by the Israeli National Health Insurance for medical indications (Israeli Ministry of Health 2021a). Women undergoing chemotherapy or radiation treatments do not need to bear the costs of fertility preservation for giving birth to up to two children (Israeli Ministry of Health 2011a). In case of increased risk for early amenorrhea, the regulations (Israeli Ministry of Health 2018) limit the funding of MEF to women under the age of thirty-nine. Funding is further limited to a maximum of four treatment cycles or twenty retrieved eggs—whichever limit is reached first (where the woman is a carrier of fragile X syndrome, the funding is limited to six cycles or forty eggs). Funding is also limited to the birth of two children of a couple (with no children in their current marriage), as well as to women without children, and to female adolescents and girls for the purpose of fertility preservation. The duration of the funded storage period is limited to the birth of two children or until the woman reaches the age of forty-two (the earlier of the two) (ibid).

In contrast, SEF is not covered by the Israeli “National Health Basket.” However, one Israeli HMO (Health Maintenance Organization)—“Meuhedet”—offers a partial subsidization of the costs for women holding supplemental medical insurance (Meuhedet 2020).

The usage of the frozen eggs at a later stage can be funded as part of the public funding scheme for IVF. Every woman aged eighteen to forty-five is in principle entitled to almost unlimited funded treatment up to the birth of two living children. Funding is not conditional to familial status and/or sexual orientation. In 2014, some moderate restrictions were issued with regard to the provision of IVF (e.g., if after eight cycles no conception occurred, a reassessment should be performed before continuing further treatment) (Israeli Ministry of Health 2021b; Israeli National Health Insurance Law 1994).

The Israeli SEF legislation also sets limitations on the *usage* of the procedure. The regulation of SEF allows freezing of eggs of healthy women aged thirty to forty-one. It further limits the procedure to a maximum of four treatment cycles or twenty retrieved eggs (the earlier of the two) (Israeli Ministry of Health 2011a). In any case, the implantation of fertilized eggs is allowed until the age of fifty-four. Eggs are stored for a five-year period, which can be extended (ibid).

In the case of MEF, as presented earlier, there are several age limitations regarding the funding of the procedure, but those focus on funding and not on usage. These differences can also be interpreted as representing a hierarchy of MEF over SEF.

#### The Netherlands

While the Dutch framework allows the performance of both MEF and SEF, an examination of the funding regulation clearly reflects the legitimation of MEF over SEF. According to the regulations, MEF is funded as part of the basic health insurance package (van der Meer and Derksen 2012). Coverage includes ovarian stimulation, oocyte extraction and cryopreservation, and medications. The potential future use of the preserved eggs might be covered if the intended woman or couple meet the requirements as set out in the current IVF regulations. Currently, the Dutch national health insurance (“Basisverzekering”) covers a maximum amount of three IVF/ICSI treatments until the woman reaches the age of forty-three (Paauw 2016). In contrast, SEF is not covered by the insurer (NVOG and KLEM 2010 2018).

In terms of eligibility criteria for egg freezing, the Dutch regulatory framework, like the Israeli one, differentiates between MEF and SEF. For social egg freezing, eggs can be retrieved and frozen for women until the age of forty (AMC 2018) and implanted until the age of fifty. In addition, women intending to undergo the procedure need to seek consultation and can be declined access to the procedure if the responsible consultant and/or treating physician see valid reason to do so.

In summary, our findings uncover the boundaries between MEF and SEF as emerged in the four countries. The boundaries are reflected and represented by certain restrictions on the very performance of the procedure as well as in selective funding (of both the procedure itself as well as future IVF), both of which significantly vary between the four countries. The specific parameters along which these boundaries are created include age, medical criteria/indications (which vary both in the level of elaboration and clarity as well as the specific medical indications included [i.e., oncological and/or fertility related]), and to some extent also marital status.

For a comparative analysis of regulatory entitlement via relevant hypothetical case studies, see table 2.

**Table 2:** Comparative analysis of regulatory entitlement via relevant hypothetical case studies

Case Study	Austria	Germany	Israel	The Netherlands
A healthy 31-year-old woman	Egg freezing is not allowed.	Egg freezing is allowed. No Funding of egg freezing will be provided. Possible partial funding of IVF.	Egg freezing is allowed (up to 4 cycles or 20 retrieved eggs). No funding of egg freezing will be provided. Funding of IVF.	Egg freezing is allowed (after social assessment in the respective clinic). No funding of egg freezing will be provided. Partial funding of IVF.
A healthy 25-year-old woman	Egg freezing is not allowed.	Egg freezing is allowed. No Funding of egg freezing will be provided. Possible partial funding of IVF.	Egg freezing is forbidden (needs to wait until the age of 30).	Egg freezing is allowed (after social assessment in the respective clinic). No funding of egg freezing will be provided. Partial funding of IVF.
A 28-year-old woman medically diagnosed as an oncological patient	Likely to be recognized as MEF and therefore allowed. No funding of egg freezing will be provided. Possible partial funding of medication and/or IVF.	Egg freezing is allowed, clearly defined as MEF and therefore fully funded, including preservation costs. Possible partial funding of IVF.	Egg freezing is allowed, clearly defined as MEF and therefore fully funded, including preservation costs. Funding of IVF.	Egg freezing is allowed, clearly defined as MEF and therefore fully funded, including preservation costs. Partial funding of IVF
A 30-year-old woman medically diagnosed with early menopause/ amenorrhea	Unclear whether will be recognized as MEF and allowed. If yes, no funding of egg freezing will be provided. Possible partial funding of medication and/or IVF.	Egg freezing is allowed. Not likely to be recognized as MEF, therefore no funding of egg freezing will be provided. Possible partial funding of IVF.	Egg freezing is allowed, clearly defined as MEF and therefore funded (up to 4 cycles or 20 retrieved eggs, until the birth of two children). Preservation costs are also funded until the birth of two children or the woman reaches the age of 42. Funding of IVF.	Egg freezing is allowed, likely to be defined as MEF and therefore fully funded, including preservation costs. Partial funding of IVF.

## Discussion and Conclusion

Rapid developments in assisted reproductive medicine are challenging decision-makers across countries. Their approaches—and hence boundaries—have far-reaching implications.

As our findings reveal, attempts at regulating egg freezing and the different regulatory boundary-work between the “medical” and “social” result in rather blurred demarcation lines. These blurred lines can be detected in two levels: The first is within each country—most prominently in Austria and in Germany, where the definition of MEF and its distinction from SEF is less clearly defined. The second level emerges from our comparative framework, which highlights cross-national differences in the regulatory frameworks. The different definitions of “medical” egg freezing and the diagnosed conditions under which egg freezing will be classified as MEF or SEF highlights the subjectivity of these definitions. At the same time, however, the way in which these terms are conceptualized has clear-cut consequences: crucial decisions, such as general access to the technology and its reimbursement, depend on these notions.

As our comparative findings show, while the four countries share a form of differentiation and prioritization when it comes to MEF and SEF, their respective regulatory frameworks also dramatically differentiate. The Austrian regulation of egg freezing is rather restrictive as it prohibits the usage of SEF and does not provide funding for MEF. While this regulation sets a crucial distinction between MEF and SEF, it does not establish any clear criterion for defining MEF. This lack of clarity reflects on the one hand a difficulty in setting the boundaries between the “medical” and the “social,” which is to some extent also reflected in the German framework. On the other hand, it also enables a legal grey zone for gatekeepers and flexibility in terms of making case-by-case decisions. The regulation in Germany has been missing to a large extent, with recent changes in the funding scheme, now also covering MEF for a relatively limited number of indications. In contrast, the Dutch framework sets much clearer definitions of the indications that account for MEF, which could, however, also be limiting considering indications that are not included in this categorization. The regulation further grants public funding for MEF, while creating a prioritization of MEF over SEF. The Israeli framework is even more detailed as it presents a clear and wide spectrum of clinical and diagnostic criteria for MEF, as well as a relatively generous funding scheme. Yet also here, funding is offered only for MEF, thus prioritizing the practice over SEF.

While we do not aim at determining which of the regulatory framework is most appropriate, our findings, hinting at a rather paradoxical dynamics involved in this “regulatory boundary-work,” should inform policymakers and their considerations. As our research reveals, a clear distinction between the “medical” and the “social” provides on the one hand transparency and distinct guidelines in daily practice, yet on the other hand may in certain cases also limit access to pre-defined indications, leading to lack of flexibility. “Blurred lines,” which in certain cases characterize rather restrictive regulatory frameworks, may in practice allow for loopholes (while creating involuntary gatekeepers). Each of the regulatory frameworks can thus be analysed as both enabling and disabling. What may seem as a restrictive and distinctive regulatory framework may paradoxically give “shelter” to SEF—be it implicitly. This in turn offers new insights into the working of the moral normativity of this distinction.

It is important to note, however, that in an ever more globalized landscape, these diverse regulatory frameworks may nevertheless contribute towards cross-border reproductive travel. Official data and even estimations of the scope are unavailable. It can be assumed, however, that reproductive travel for the purpose of egg freezing is a seriously considered option, particularly for women living in countries where access is restricted.

Furthermore, the differentiation between “medical” and “social,” even though used as starting point for regulatory decisions, is not self-evident and reflects very different and diverse boundary-work. Our findings therefore, present an important added value to previous bioethical research and literature discussing the problematization around the artificiality of this distinction (see e.g., Martin 2010; Pennings 2013; Van de Wiel 2015).

The difficulty in defining MEF, and the diverse regulation when it comes to including different genetic and chromosomal indications and other infertility related conditions, evolved as a specific regulatory difference in our findings. Such a practical challenge is of particular relevance to the bioethical debate focusing on the connection between SEF and (bio)medicalization—raising the question of whether age-related fertility decline should be regarded as a medical condition/problem justifying the usage of medical means. Within this context, SEF was controversially regarded as a form of preventive medicine (Shkedi-Rafid and Hashiloni-Dolev 2011 and 2012; Martin 2010).

In general, the regulatory frameworks we explored clearly indicate a prioritization of “medical” over “social” motivations. In this context, age related fertility decline per se is not classified as a medical reason for fertility preservation. Furthermore, the partly stringent description of medical indications also prioritizes some medical reasons over others. For example, while in all four countries oncological treatments were identified as qualifying for MEF, other conditions leading to infertility (e.g., chromosomal and genetic disorders) are officially acknowledged only by the Dutch and Israeli regulations.

Another important point to be noted are the cross-national differences in terms of access and funding and the potential future use of the frozen eggs via IVF. While Israel provides women with more possibilities to use the frozen eggs (regardless of whether they were frozen for “social” or “medical” reasons) due to its rather generous funding policy, the Austrian and German funding schemes are much more limiting. The selective funding scheme in Germany and Austria of the usage of IVF by single women set yet another boundary which is of relevance specifically for SEF (Kostenzer 2020). In Israel and in the Netherlands, single women are allowed to freeze their eggs for “social” reasons, knowing that they will receive funding when wishing to use those eggs via IVF regardless of their marital status. In contrast, in Germany, single women can use SEF but will not receive funding for IVF, and in Austria SEF is forbidden altogether. These differences may also hint towards different biopolitical approaches and perceptions of the “appropriate” family model across the countries, which, however, require further investigation.

Another implication identified, are existing age restrictions. Such boundaries should be understood in light of the related medical risks and pregnancy complications at older maternal ages for both the mother as well as the future offspring (for relevant discussion, including counter arguments see Bernstein and Wiesemann 2014; Baldwin et al. 2014). Furthermore, by setting boundaries for assisted reproduction, scarce resources in healthcare are rationed, also based on health technology assessment and efficacy. At the same time, however, these limitations might unintentionally contribute towards normative indications of what constitutes the “appropriate” maternal age. SEF may challenge normative ideas regarding the appropriate life course and reproductive age norms (Rimon-Zarfaty and Schweda 2019; Baldwin et al. 2014). Regulatory frameworks might therefore seemingly and implicitly also reinforce social normative ideals of women’s biographies and reproductive timing. Indeed, in all four countries, we observed some form of age limitation regarding IVF funding regulation and in Israel, Germany and the Netherlands also regarding egg freezing usage (in the context of SEF) and/or funding (in the context of MEF).

The variation and blurring we detected in our research may further reflect certain underlying norms, one of which may include ageism. Observed age restrictions may reflect certain controversial ethical concerns regarding women’s physical and mental capability of becoming mothers at older ages as well as the potential social and emotional difficulties for children of older parents (for critical discussion see Bernstein and Wiesemann 2014; Baldwin et al. 2014). Ideas regarding old parenthood are also highly gendered and thus may be critically interpreted as form of sexism while highlighting the “double standard of aging” (Sontag 2018) in the context of sexuality (Pickard 2016) as well as the moral assessment of late parenthood (Bernstein and Wiesemann 2014; Rimon-Zarfaty and Schweda 2019). With regard to egg freezing regulation, those may be reflected in the different age limitations which differentiate between men and women. Cross-national differences in boundaries apparent in funding and accessibility policies may also be understood in the context of pronatalism. In relation to Israel for example, the overall favourable regulatory framework of fertility medicine and preservation can be understood in the context of pronatalist culture (Shkedi-Rafid and Hashiloni-Dolev 2011). The cross-national differences in the regulatory frameworks may also be analysed in the context of geneticism and the importance of genetic motherhood. Indeed, in the ethical debate on egg freezing, the possibility of genetic/biological motherhood at advanced maternal age was discussed as one of the technology’s main advantages (Petropanagos et al. 2015; Dondorp and de Wert 2009).

Our study, however, also has limitations. By comparing regulatory frameworks, we could gain valuable insights as to how ideas of “medical” and “social” find practical expression. However, we did so by primarily researching documents. While we did consult experts in the field, a more in-depth approach of the respective stakeholders' perceptions is considered useful in getting a better understanding of the underlying intentions. Furthermore, we need to be aware that our analysis focused merely on the written practice, which might differ from the actual practice, on which we are not able to draw in-depth conclusions.

In conclusion, egg freezing remains to be a controversial topic for bioethical academic debate, policy, and practice, leading to convergences and divergences in the states’ understanding regarding if and how to regulate it. Within the scope of the current paper and the nature of our research we were not able to investigate possible explanations for the cross-national differences we detected in the regulatory frameworks (e.g., possible relevant sociocultural scripts). Yet, by outlining these differences, this paper problematizes the oversimplification of both concepts (MEF and SEF) and their differentiation. By that, the abstract perspective around this differentiation is expended while unveiling cross-national differences and implicit normative perceptions. Those may be further discussed in the context of bio/cryo-political mechanisms (Radin and Kowal 2017). As life is extendedly regulated by technoscientific means, the practice of cryopreservation can be analysed as facilitating the effort to choreograph ontological state (e.g., “mother” or “parent”) (ibid,13). Following our research, we wish to highlight the way such efforts, with their sociocultural underpinnings, can be reflected in and uncovered by the different regulatory frameworks. Exploring ethical and biopolitical aspects further could help to better understand regulatory differences and decision-making processes, while supporting the identification of the relevant normative implications.
